# Primary Cardiac Angiosarcoma: A Rare and Fatal Diagnosis

**DOI:** 10.7759/cureus.20816

**Published:** 2021-12-29

**Authors:** Inês Gonçalves, Catarina Nunes, Catarina Vieira, Diana Freitas, Luisa Pinto

**Affiliations:** 1 Internal Medicine, Hospital de Braga, Braga, PRT; 2 Cardiology, Hospital de Braga, Braga, PRT; 3 Oncology, Hospital de Braga, Braga, PRT

**Keywords:** pulmonary metastases, cardio-oncology, transthoracic echocardiogram, pericardial effusion, cardiac angiosarcoma

## Abstract

Primary cardiac angiosarcoma is a rare malignant neoplasm and occurs most frequently in middle-aged males. It has an aggressive nature, with highly variable clinical features, which results in delayed diagnosis and high mortality.

We report a 19-year-old man presented to the ED with a three-month history of hemoptysis and one-week history of anterior chest pain. Additionally, an aortic diastolic murmur grade II/VI was found on physical examination. Thoracic CT scan revealed bilateral dispersed hypodense pulmonary nodes with peripheral halo, alveolar densification, and pericardial effusion. The transthoracic echocardiogram confirmed sizeable pericardial effusion and bicuspid aortic valve, without other significant findings. A pericardiocentesis removed 1300 mL of hemorrhagic fluid, consistent with an exudate without malignant cells. Both cardiac magnetic resonance and transesophageal echocardiogram revealed a large mass on the right atrium’s anterior wall. Mass biopsy was performed, revealing malignant cardiac angiosarcoma. The biopsy of the lung lesions was compatible with lung metastasis of primary cardiac angiosarcoma. The patient was submitted to palliative chemotherapy but died 12 months after the diagnosis.

## Introduction

Primary cardiac angiosarcoma, although extremely rare, is the most common primary malignant cardiac neoplasm [1, 2]. It is a life-threatening entity with a poor prognosis due to its extraordinarily aggressive behavior, even when complete resection of the tumor is performed [2, 3]. The symptoms' late-onset and insidious development contribute to a diagnostic delay. Approximately 80% of cardiac angiosarcomas present metastatic spread at the time of diagnosis, which restricts the surgical resection indication to a small number of affected patients [4]. We report a case of a metastatic primary cardiac angiosarcoma in a very young male, complaining of chest pain and hemoptysis. Despite the advanced stage of disease, this case reflects the late onset of symptoms, disease severity, and the complexity of its diagnosis.

## Case presentation

**Figure 1:**
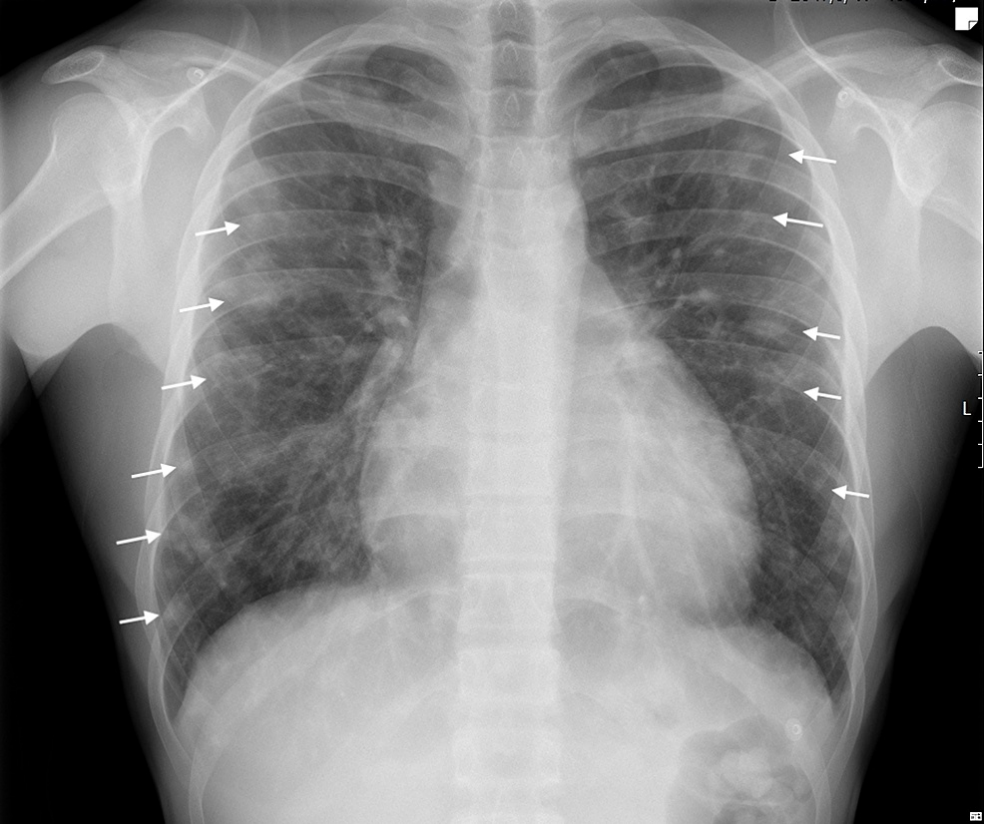
Chest radiograph (posteroanterior view). Multiple, bilateral, nodular opacities of variable size (white arrows) and cardiomegaly.

A 19-year-old man presented to the ED with a one-week history of anterior chest pain. The pain was non-radiating and increased in intensity with deep breathing, without other exacerbating or relieving factors. He also described small hemoptysis during the last three months. The patient denied dyspnea, asthenia, fatigue, anorexia, weight loss, fever, night sweats, or the presence of peripheral lymphadenopathies. His medical history included asthma in childhood and chronic sinusitis. However, he did not take regular medication. His physical examination was unremarkable, except for an aortic diastolic murmur grade II/VI on cardiac auscultation. Laboratory tests revealed a hemoglobin concentration of 11.3 g/dL (reference range: 13.5-17.0 g/dL), white cell count of 12.9 × 109/L (reference range: 4.0-11.0×109/L), and platelet count of 590 x 109/L (reference range: 150-400×109/L). Inflammatory markers were elevated, with an erythrocyte sedimentation rate of 60 mm (reference range: <25 mm/h) and a C-reactive protein of 90.6 mg/L (reference range: <5 mg/L). The NT-proB-type natriuretic peptide was negative and cardiac enzymes levels were normal. The 12-lead ECG presented normal sinus rhythm, with a normal axis and no Q wave or ST-T changes. Chest radiograph showed mild cardiomegaly and bilateral nodular infiltrates (Figure 1).

**Figure 2:**
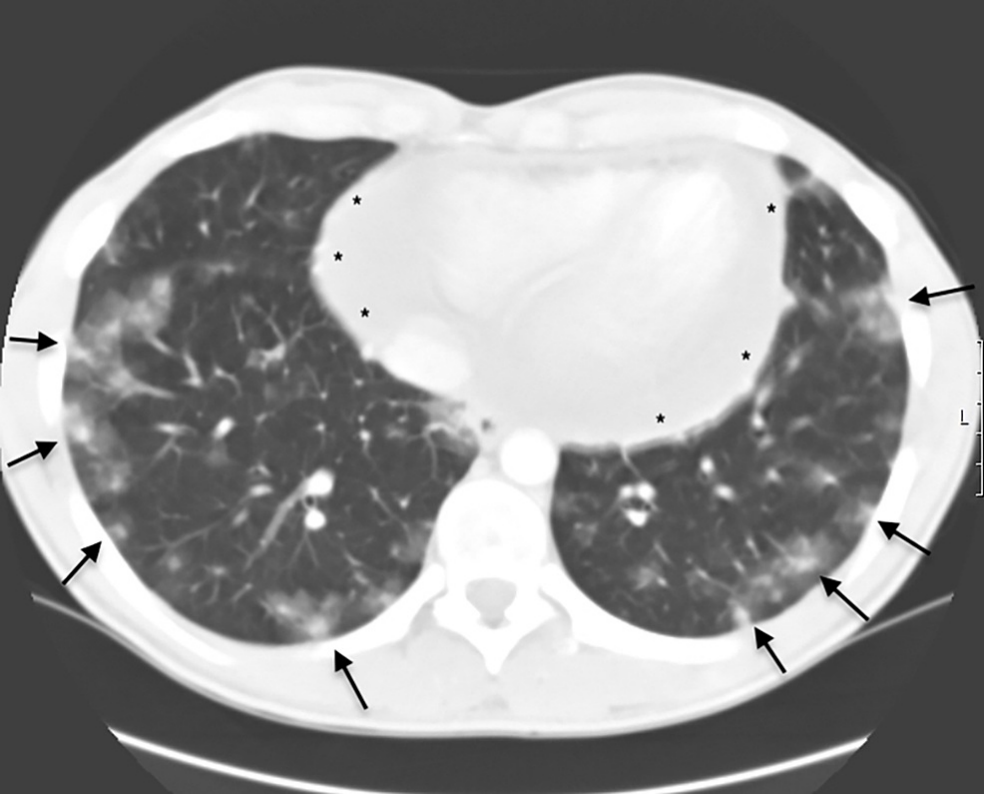
Thoracic CT scan. Thoracic CT demonstrated bilateral dispersed hypodense pulmonary nodes with peripheral halo, alveolar densification (arrows), and a large pericardial effusion (*).

This report was generated prior to the pandemic of COVID-19, so a PCR for SARS‑CoV‑2 was not undertaken. A thoracic CT scan was performed, which revealed bilateral dispersed hypodense pulmonary nodes with peripheral halo and alveolar densification. A large pericardial effusion was also seen. However, no masses were found (Figure 2). 

Transthoracic echocardiography disclosed a standard cardiac size and ventricular function, a bicuspid aortic valve with mild aortic insufficiency, and large pericardial effusion (35 mm on the free wall of the right ventricle in subcostal view) with no tamponade features or signs of obstruction to blood flow. A pericardiocentesis procedure was performed, removing 1300 mL of hemorrhagic pericardial fluid. Laboratory analysis showed an exudate. The cytological analysis did not show evidence of malignancy. Cultural tests of pericardial fluid were negative for bacteria, including Mycobacteria. An abdominal and pelvic CT scan was performed without remarkable findings.

**Figure 3:**
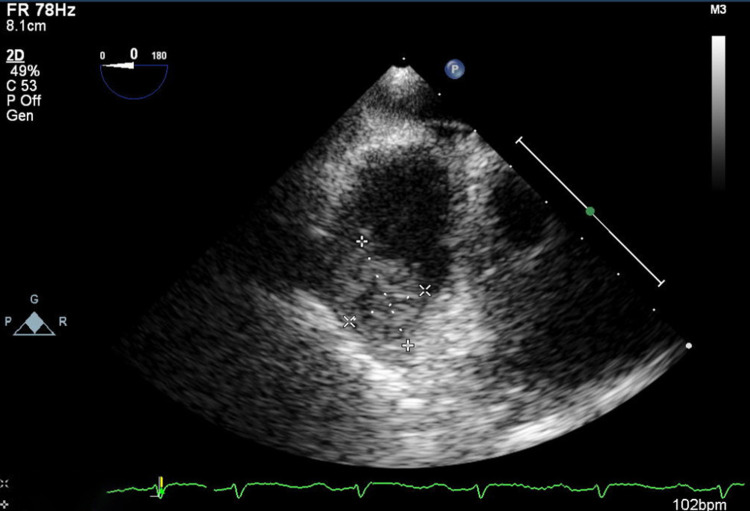
Transesophageal echocardiogram. Sessile heterogeneous irregular mass of 32 x 18 mm occupying the right atrium.

Further evaluation included bronchoscopy, which demonstrated dispersed blood over the bronchial tree without active or recent hemorrhage. Bronchoalveolar lavage and lung biopsy were inconclusive and did not help to establish a definite diagnosis. Viral serology tests for HIV, hepatitis B virus (HBV), hepatitis C virus (HCV), and syphilis were negative. Autoimmune screening, including antinuclear antibodies (ANA), double-stranded DNA antibodies (dsDNA), antineutrophil cytoplasmic antibodies (ANCA), anti-glomerular basement membrane antibody (anti-GMB), was negative. The beta subunit of human chorionic gonadotropin (β-hCG) and alpha-fetoprotein were also negative. In addition, bacterial and fungal blood cultures were negative. The paranasal sinus CT scan found a thin inflammatory mucous thickening without further changes. A nasal turbinate biopsy was performed to exclude a granulomatous vasculitis, and the histological result did not reveal any characteristic finding. A transesophageal echocardiogram was made to further proceed with the investigation and demonstrate a sessile heterogeneous mass of approximately 32 x 18 mm with an irregular shape occupying the right atrium (Figure 3).

**Figure 4:**
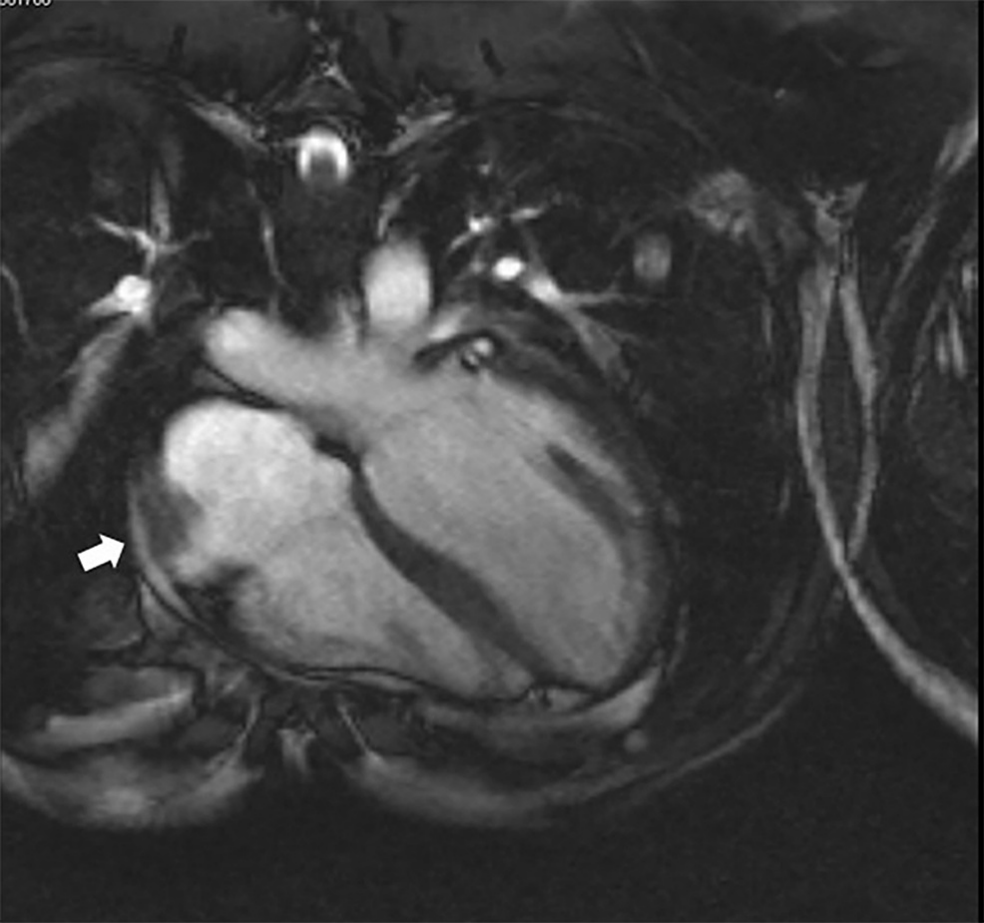
Cardiac MRI. Mass of 8 x 3.5 x 2 cm at the anterior wall of the right atrium (arrow).

For better characterization of the mass, a cardiac magnetic resonance was performed. It revealed a mass of 8 x 3.5 x 2 cm at the anterior wall of the right atrium, extending from the anterior slope of the superior vena cava and ascending aorta, being in contact with these structures in less than 50% of their circumference (Figure 4).

The finding was highly suspicious of an intracardiac primary malignant tumor. The patient was finally referred to a health care center with a cardiothoracic surgery department to achieve a biopsy of the right atrium. Histology of the mass suggested a primary malignant cardiac angiosarcoma. In order to establish the prognosis and select the most suitable treatment, a biopsy of the lung lesions was made by conventional thoracic surgery. The result was compatible with lung metastasis of primary cardiac angiosarcoma. 

The patient began palliative chemotherapy with paclitaxel. Unfortunately, he initiated diplopia, nausea, and vomiting during treatment, suggesting cerebral metastasis, later confirmed by head CT. Twelve months after the initial diagnosis, the patient died.

## Discussion

Primary cardiac neoplasms are rare clinical entities with an autopsy incidence of 0.001%-0.3% [3, 5, 6]. About 25% of these tumors are malign, represented mainly through cardiac sarcomas. Although extremely rare, primary cardiac angiosarcoma is the most common histological subtype. These tumors have been identified among many ages, but patients are predominantly male between the third and fifth decade of life [7, 8]. Around 75% of angiosarcomas are located in the right atrium. The lateral/free wall is the most common site, while the interatrial septum is frequently preserved [4, 7].

The signs and symptoms usually begin gradually and can be absent for a long time [4, 5]. They may be nonspecific or related to the tumor’s location, the affected area’s size, or the presence or absence of metastasis [9].

The most frequent symptoms relate to right heart failure, as the right atrium is the most common location [9, 10]. Dyspnea is present in 59%-88% of patients with such a diagnosis [2, 7]. It is usually multifactorial: vulnerability of the tricuspid valve due to the location in the right atrium, pericardial effusion with or without cardiac tamponade, right ventricle obstruction due to tumor’s growth, pleural effusion, and lung metastasis [4, 9, 11, 12]. Less frequently, patients may present hemopericardium, hemoptysis, anemia, pulmonary embolism, and superior vena cava syndrome [10, 12].

Imaging techniques such as transthoracic or transesophageal echocardiogram are crucial to evaluate and locate cardiac tumors [2, 10-12]. In addition, they are effective and minimally invasive procedures [11, 13].

As an initial diagnostic test, transthoracic echocardiography has a sensitivity of 75% for visualizing primary angiosarcoma [11]. However, its sensitivity may decrease in the presence of pericardial effusion [2]. Therefore, it is essential to maintain clinical suspicion in the case of pericardial effusion, even if no cardiac masses are found. On the other hand, the transesophageal echocardiogram has a 97% sensitivity identifying cardiac masses [14, 15]. Therefore, it is also essential in the follow-up post-cardiac surgery (when surgery is recommended) [11].

Other imaging techniques such as CT and cardiac MRI also contribute to an early diagnosis [6]. In addition, they are crucial in determining the disease’s extension and defining extracardiac invasion and metastasis [11, 16]. Cardiac MRI is the most widely used imaging method to characterize a cardiac mass, and the addition of contrast permits a better characterization of the tumor and its vascularization. In addition, it can suggest a tissue diagnosis [12, 16]. Nevertheless, the final diagnosis is given through the histological result.

Pericardium involvement is frequent. Although pericardiocentesis, when performed, often reveals bloody effusion, the cytological examination is rarely conclusive, even in the presence of significant pericardial tissue involvement [7, 8]. Consequently, a normal result of the pericardial effusion does not exclude malignancy [11].

An early diagnosis of this tumor is a challenge due to its insidious behavior [4]. Most of the time, it is not done promptly, so between 66% and 89% of patients have metastasis at the time of diagnosis [4, 7]. Its aggressive behavior is reflected in the mean survival of 6-12 months after establishing the diagnosis [4, 10, 12]. Treatment options are related to the aggressive nature of the tumor and the presence or absence of tumor dissemination [12, 17]. 

In the absence of metastasis, surgery to remove tumor mass remains the first-line treatment [3, 4, 7, 13]. In the presence of disseminated disease, surgical resection is not curative, but it may be advantageous to reduce the associated symptoms [5]. As a result, early diagnosis improves the possibility of complete surgical removal of the tumor and its prognosis [9]. Additional treatment is not established due to the rarity, but multidiscipline therapies, including radiotherapy and chemotherapy, are most commonly used [17]. Further in-depth studies are needed to demonstrate the role of each therapeutic method and outcome improvement. 

Heart transplantation has been performed on rare occasions in patients with primary cardiac angiosarcoma, in most cases without survival benefits. In fact, it seems that immunosuppressive drugs may be associated with increased tumor growth and spreading [18]. The leading causes of death are myocardial invasion, cardiac tamponade, metastasis, embolism, and arrhythmias [13].

## Conclusions

Cardiac angiosarcoma, although extremely rare, is the most common malignant primary cardiac sarcoma. The signs and symptoms may be nonspecific, usually begin gradually, and frequently derive from tumor metastasis. Maintaining a high level of suspicion is crucial, especially when a young patient presents with nonspecific long-lasting symptoms such as dyspnea, which is the most frequent symptom. An early diagnosis is difficult but crucial to improve survival. In most cases, transthoracic echocardiography and CT are central for establishing the diagnosis. However, in the presence of large-volume pericardial effusion, they can be non-diagnostic, and the tumor can go unnoticed. Thus, performing a transesophageal echocardiogram or cardiac MRI may be necessary. The histological result gives the final diagnosis. Unfortunately, due to the aggressive nature of the tumor, the prognosis is very poor (mean survival of 6 to 12 months after diagnosis establishment) even with all the therapeutic options (surgery, chemotherapy, radiation therapy, and immunotherapy).
